# Quantum Hooke's Law to Classify Pulse Laser Induced Ultrafast Melting

**DOI:** 10.1038/srep08212

**Published:** 2015-02-03

**Authors:** Hao Hu, Hepeng Ding, Feng Liu

**Affiliations:** 1 Frontier Institute of Science and Technology, Xi'an Jiaotong University, Xi'an710054, China; 2 Department of Materials Science and Engineering, University of Utah, Salt Lake City, UT84112, USA

## Abstract

Ultrafast crystal-to-liquid phase transition induced by femtosecond pulse laser excitation is an interesting material's behavior manifesting the complexity of light-matter interaction. There exist two types of such phase transitions: one occurs at a time scale shorter than a picosecond via a nonthermal process mediated by electron-hole plasma formation; the other at a longer time scale via a thermal melting process mediated by electron-phonon interaction. However, it remains unclear what material would undergo which process and why? Here, by exploiting the property of quantum electronic stress (QES) governed by quantum Hooke's law, we classify the transitions by two distinct classes of materials: the faster nonthermal process can only occur in materials like ice having an anomalous phase diagram characterized with dT_m_/dP < 0, where T_m_ is the melting temperature and P is pressure, above a high threshold laser fluence; while the slower thermal process may occur in all materials. Especially, the nonthermal transition is shown to be induced by the QES, acting like a negative internal pressure, which drives the crystal into a “super pressing” state to spontaneously transform into a higher-density liquid phase. Our findings significantly advance fundamental understanding of ultrafast crystal-to-liquid phase transitions, enabling quantitative *a priori* predictions.

Light-matter interaction, such as pulse laser induced ultrafast dynamics of matter is an important topic in materials science and condensed matter physics. Upon pulse laser excitation, a high density of electrons are excited, forming a highly non-equilibrium state that drives an ultrafast dynamic responses of electrons, atoms and lattice to the photon energy. The earlier experiments, based on optical method^1^,^2^,^3^,^4^,^5^,^6^,^7^,^8^,^9^, probe the ultrafast response of electrons to photons, while the change of atomic and lattice structures is inferred from the changing optical properties measured from reflectivity and second-harmonic generation, etc.; recent development of time resolved x-ray and electron diffraction enables an *in situ* probe of the ultrafast atomic and lattice dynamics^10^,^11^,^12^,^13^,^14^,^15^,^16^,^17^,^18^,^19^,^20^,^21^.

Structural phase transitions induced by pulse laser excitation have attracted much interest for both fundamental understanding and practical applications. An interesting phenomenon is the pulse laser induced “melting” or crystal-to-liquid phase transition which has been exploited in optical storage devices^22^. In general, it is found there are two types of such phase transitions differing by time scale: one occurs faster [< ~1 picosecond (ps)] and the other one slower (> ~1ps). The faster transition is a nonthermal process, mediated by electron-hole plasma formation^1^,^2^,^3^,^4^,^6^,^7^,^8^,^9^,^12^,^13^,^14^,^15^; while the slower transition is understood in terms of conventional thermal melting process, mediated by electron-phonon interaction^1^,^2^,^19^.

A fundamental gap in our understanding of the pulse laser induced crystal-to-liquid transition lies in the fact that it is unclear what ‘signature’ property defines a material's melting behavior under pulse laser irradiation. As a result, it remains *a priori* unknown which material would undergo faster or slower melting process. The slower melting process is a thermal melting process, occurring in a time scale of several ps (or longer) in the same order of the electron-phonon thermal equilibration time; it can induce crystal-to-liquid phase transition in all types of materials. The faster melting process must be a nonthermal process, because it occurs in a time scale shorter than the electron-phonon equilibration time. Experiments have observed the faster nonthermal melting process in some semiconductors (e.g. Si^3^,^4^,^5^,^11^, Ge^10^, GaAs^6^,^7^,^8^,^9^ and InSb^12^,^13^,^14^) and semimetals (e.g. Bi^15^,^16^,^17^,^18^) at high laser fluence. Also in these semiconductors and semimetals, the slower thermal melting is observed at low laser fluence. In contrast,in some fcc metals (e.g. Au^19^, Al^20^ and Ag^21^), only the slow thermal melting is observed. However, the fundamental difference in material properties that are responsible for the different melting behavior is unknown.

The thermal melting process is relatively well understood. The photo-excited hot electrons transfer their kinetic energy to atoms by activating phonons via electron-phonon interaction and increase lattice temperature above the melting point. In contrast, the physical mechanism underlying the nonthermal melting process is much less understood. The phase transition occurs within hundreds of femtoseconds (fs), so that the electrons remain hot while lattice stays cold during the transition. The transition is known to be associated with the electron-hole plasma consisting of excited electrons and holes. It was suggested that the excited electrons might soften and eventually break the interatomic bonds to form a disordered phase^1^,^2^,^3^,^6^,^11^,^15^. But the defining physical parameter of the electron-hole plasma that controls the nonthermal melting process, similar to temperature that controls the thermal melting process, remains unknown.

In this Article, by exploiting the property of QES^23^, we achieve a more comprehensive understanding of the pulse laser induced crystal-to-liquid transition. Most importantly, we identify that the nonthermal melting process can only occur in a class of materials like ice having an anomalous phase diagram with dT_m_/dP < 0, based on the nature (sign) of the QES generated by the electron-hole plasma. The transition occurs by the QES acting like a negative internal pressure that drives the crystalline phase into a “super pressing” state to spontaneously transform into a higher-density liquid phase at low temperature. This key identification allows us to rationalize all the known materials that exhibit the crystal-to-liquid transition under pulse laser irradiation in experiments^1^,^2^,^3^,^4^,^5^,^6^,^7^,^8^,^9^,^10^,^11^,^12^,^13^,^14^,^15^,^16^,^17^,^18^,^19^,^20^,^21^, as well as to predict unknown materials that can undergo the nonthermal melting process. Moreover, the QES is shown to be predominantly induced by holes, rather than the excited electrons, which increases linearly with the hole density following the quantum Hooke's law^23^. This finding enables us to further quantitatively estimate the threshold laser fluence required for the non-thermal melting process in different materials, consistent with all the available experiments^1^,^2^,^3^,^4^,^5^,^6^,^7^,^8^,^9^,^10^,^11^,^12^,^13^,^14^,^15^,^16^,^17^,^18^.

We first review the concept and property of QES^23^. QES is recently introduced to elucidate the extrinsic electronic effects on the stress state of a solid in the absence of lattice strain. Conventionally, the lattice stress in a solid is considered as mechanical stress (σ^M^) arising from lattice strain (*ε*), following the classical Hooke's law, *σ^M^* *= Cε*, where *C* is elastic modulus. Now consider an equilibrium lattice in the absence of strain (*ε = 0*), but electronically perturbed or excited, such as an electron is kicked out by a photon leaving behind a hole^23^, that redistributes the electron density. The associated “electronic deformation” energy, in an adiabatic approximation, can be expressed as *ΔE = μΔN*, where *μ* is the electron chemical potential and *ΔN* is the change of number of electrons. Then the lattice stress induced by such electronic deformation, which is referred to as QES (*σ^QE^*), can be expressed in quantum Hooke's law as *σ^QE^* *= ΞΔn*, where *Ξ = dμ/dε* is the deformation potential and *Δn* is the change of electron density. A rigorous mathematic formulation of QES within density functional theory (DFT) has been given in ref. 23.

In a pulse laser experiment, initially photoexcitation forms a high-energy electron-hole plasma^1^,^2^,^3^,^24^ before electron-phonon thermal equilibration. In the electron-hole plasma, the valence holes are relatively localized due to their lower mobility; while the nearly free hot electrons are more diffuse losing their coherence with the holes very shortly (within 100 fs)^1^, which can be effectively treated as a homogeneous electron gas. Generally, electron mobility (μ) and diffusivity (D) are a few times up to two orders of magnitude larger than hole mobility and diffusivity in semiconductors at same temperature^25^. In addition, the excited electrons have a much higher temperature (T = 10000 K–20000 K) than holes (~300 K), which makes the electron diffusivity further higher following Einstein relation (D = μk_B_T). Because formation of electron-hole plasma occurs in all materials with similar plasma characteristics, i.e. material independent, in a pulse laser experiment, we will apply this approximate treatment to all the materials in this study. Then we can calculate the QES induced by the electron-hole plasma by adding the contribution of the holes and the electron gas separately. We have performed DFT calculations of QES induced by the localized holes, as shown in Fig. 1, and electron gas as a function of temperature [Supplementary Information] in electron-hole plasma. In a typical pulse laser experiment, the excited electron density is up to 2 × 10^22^ cm^−3^. Our calculations show that at such density, the QES generated by the homogeneous electron gas is negligible compared to that generated by the holes of the same density [see Fig. 1 and Supplementary Information]. Thus, the QES associated with the electron-hole plasma is dominated by the QES induced by the holes.

Next, we postulate that the nonthermal melting process is driven by the QES acting like pressure, so that the pulse laser induced ultrafast (< ~1 ps) crystal-to-liquid transition can be effectively treated as a *non-equilibrium super-pressing transition at low temperature*. If so, we show that it can only occur in materials like ice having an anomalous phase diagram with dT_m_/dP < 0 and above a threshold laser fluence. It has been shown that the holes (electrons) always generate a tensile (compressive) QES, tending to shrink (expand) the lattice, respectively^23^. This means that the overall QES of electron-hole plasma, dominated by the hole's contribution, is tensile, which will induce effectively a negative internal pressure to shrink the lattice during the crystal-to-liquid transition. Consequently, the resulting liquid phase must have a higher atomic density (smaller atomic volume) than the crystalline phase, similar to ice that shrinks upon melting into water. We have checked the phase diagrams as well as densities of the crystal and liquid phases of the known materials^1^,^2^,^3^,^4^,^5^,^6^,^7^,^8^,^9^,^10^,^11^,^12^,^13^,^14^,^15^,^16^,^17^,^18^ that undergo ultrafast nonthermal melting process under femtosecond pulse laser excitation. Indeed, they are all found to have an anomalous phase diagram, as shown in Table I. In contrast, we also checked the materials that undergo only thermal melting process under femtosecond pulse laser excitation, and as expected, they all have a normal phase diagram^19^,^20^,^21^,^28^(Table I). One interesting case is Al, which was originally thought undergoing the nonthermal melting process but later corrected to be thermal process by time resolved electron diffraction^20^. Thus, our theoretical results are in good agreement with experimental observations.

The above analysis based on the nature (sign) of QES provides a strict material classification for the ultrafast pulse laser induced crystal-to-liquid transition as illustrated in Fig. 2. Under high enough laser fluence, the nonthermal melting process, occurring within ~ 1 ps mediated by electron-hole plasma, is driven by QES and belongs to a class of materials having an anomalous phase diagram characterized by dT_m_/dP < 0. The known examples include Si, Ge, GaAs, InSb and Bi^1^,^2^,^3^,^4^,^5^,^6^,^7^,^8^,^9^,^10^,^11^,^12^,^13^,^14^,^15^,^16^,^17^,^18^. The thermal melting process, occurring beyond ~ 1 ps mediated by phonon cloud, is driven by temperature which may occur in all materials at relative lower laser fluence.

Next, we perform a quantitative analysis of the threshold hole density and hence the threshold laser fluence, above which the nonthermal melting process occurs. As discussed above, the QES acts like a negative internal pressure, or equivalently we can think of the surrounding crystal lattice applying a (positive) pressure of the same magnitude as QES to the laser illuminated region within which the electron-hole plasma forms. The QES rises very quickly within the duration of the pulse laser; the loading rate can be so high that the excited region of crystal enters a “super pressing” state, so that the crystalline phase becomes unstable and transform spontaneously into the stable liquid phase via a highly non-equilibrium process. If so, using our calculated QES and the solid-liquid phase diagram, we can estimate the minimum pressure needed to reach the "super pressing" state at room temperature, and the corresponding electron-hole plasma density, and then compare them with the experimental threshold density.

For those materials having dT_m_/dP < 0, the solid-liquid equilibrium has been measured in certain pressure range in the T-P phase diagram, for example, 0–15 GPa for Si, 0–1 GPa for Bi^28^. But the QES rises quickly to much higher pressures. Thus, to estimate the threshold density, we extend approximately the solid-liquid equilibrium line to higher pressures by linear extrapolation, as shown in Fig. 3 for Si, Ge, GaAs, InSb and Bi. Then, we estimate the minimum pressure needed for reaching the super-pressing state at room temperature, i.e., the intersection points between the arrowed solid line at room temperature and the metastable solid-liquid line. Under the super-pressing condition, the crystalline phase is unstable which will transform spontaneously into stable liquid phase. We note that theoretically the solid-liquid equilibrium line is exact linear, if the molar volume and heat capacity differences between the liquid and solid phase is independent of temperature. In reality, they are only a weak function of temperature that introduces small non-linearity at high temperature, but essentially independent of pressure. Since in our case the lattice temperature remains low, we expect that the linear extrapolation to high pressure is a reasonably good approximation. Of course, future experiment over a larger pressure range is needed to further confirm the validity of our assumption. Nevertheless, we expect this approximation causes only small quantitative errors that will not change our qualitatively conclusion, namely our predicted theoretical thresholds remain lower than the experimental values, as shown below.

The experimentally measured threshold electron-hole plasma densities vary over a certain range. Here we take some typical values for comparison: 9% valence electrons for Si^10^, 17%, 25%, 5.8% and 3% for Ge^10^, GaAs^6^,^7^, InSb^13^ and Bi^15^,^16^, respectively. From Fig. 1, the QES at these excitation levels can be obtained, as marked by stars in Fig. 3. We can see that the QES at the experimental threshold plasma densities exceed consistently the minimum theoretical pressure needed to reach the super-pressing state, indicating our theory is qualitatively correct for all the materials considered. There are some large quantitative variations between the experimental and theoretical values, which may originate from several different sources. Experimentally, there could be uncertainties in the measured threshold densities, as any given density above the threshold density may induce the phase transition but it is difficult to pin down accurately the exact threshold density. In other words, there could be a different amount of overshooting of pressure for experimentally inducing the super pressing transitions, similar to overshooting of temperature for super cooling transitions. There are also uncertainties in our theoretical estimation of threshold densities, arising from DFT method itself, the approximate treatment of electron-hole plasma and the linear extrapolation of solid-liquid equilibrium line as discussed above. Given these experimental and theoretical uncertainties, we consider the agreement between experiment and theory is rather satisfying. We note that the materials having dT_m_/dP < 0 that exhibit nonthermal melting at a high threshold density may also exhibit thermal melting at a lower density, which has indeed been observed in experiments^1^,^2^,^3^,^4^,^5^,^6^,^7^,^8^,^9^,^10^,^11^,^12^,^13^,^14^,^15^,^16^,^17^,^18^. In contrast, for the materials with normal phase diagram of dT_m_/dP > 0, such as Au, Al and Ag^19^,^20^,^21^, they will never enter the super-pressing state by increasing pressure at room temperature, so that they can only exhibit thermal crystal-to-liquid melting transition.

Importantly, based on our theory, we can predict new materials that will exhibit nonthermal crystal-to-liquid transition for future experimental studies. Table II lists several materials predicted, including AlSb, GaSb and InAs, which have the zinc-blende structure, and Mg_2_Si, Mg_2_Sn and Mg_2_Pb, which have the antifluorite structure. We further predict the minimum pressure needed to reach "super pressing" state and calculate the QES as a function of hole density (quantum Hooke's law, see Fig. S1 in Supplementary Information) in these materials. The threshold plasma densities are 5.9%, 6.2%, 9.5%, 2.5%, 11.6%, and 4.4% for AlSb, GaSb, InAs, Mg_2_Si, Mg_2_Sn and Mg_2_Pb, respectively, which should set the lower limits for experimental observation.

We should mention that our macroscopic theory analysis, based on QES and quantum Hooke's law, does not capture microscopic atomic process and dynamics involved in the nonthermal crystal-to-liquid transition. Nevertheless, some of our general conclusions are found to be consistent with existing experimental and theoretical microscopic and dynamics studies. Given the anomalous phase diagram, naturally the atomic density of resulting liquid is larger than that of parent crystal. This is consistent with experimental observation of pulse induced lattice contraction before electron-phonon equilibration^29^. It also agrees with the finding from *ab initio* molecular dynamics simulation^30^ that the nonthermal melted Si has a much higher coordination number than that of normal liquid Si, implying a high pressure phase. Another strong evidence for the role played by QES is the internal atomic displacement that changes with the excited carrier density (or the pulse laser fluence) in Bi^17^. Bi is group-V elements with a rhombohedral crystal structure, which can be viewed as a simple cubic (SC) structure undergoing two distortions: an internal displacement of two interpenetrating fcc lattice along the (111) diagonal direction, and a trigonal shear extending along the same direction^31^. QES will suppress such distortions, forcing the internal displacement to decrease and hence promoting the phase transition from rhombohedral to SC structure, in agreement with previous DFT calculation that has shown such transition in Bi by applying compressive strain^31^. The “negative pressure” effect induced by QES is also consistent with the experiment that showed evidence of the internal displacement in Bi decreasing with the increasing excited carrier density (pulse laser fluence)^17^.

On the other hand, we stress that the QES effect cannot be simply explained by phenomenological bond theory. The bond theory, as adopted from molecular photoisomerization, would predict that when electrons are excited from bonding state to antibonding state, the bonds are softened so that the interatomic interaction becomes repulsive, giving rise to a positive internal pressure^32^ which tends to expand the lattice. However, this argument is not generally applicable to the solid. Our QES theory shows that upon pulse laser irradiation, the photoexcitation induced electron-hole plasma exerts a tensile QES, equivalent to a negative internal pressure, which tends to shrink the lattice. Previous study also suggests that the electron gas and the atomic lattice are coupled through the exchange of mechanical work^15^,^32^, albeit implying a positive internal mechanical pressure to weaken the bond that would be associated with a lattice expansion. However, we propose that the mechanical work exchange is done by a tensile QES to shrink (super pressing) and collapse the lattice. In particular, the QES will induce large atomic displacements, including those transverse to the force direction due to the tetrahedral-coordinated bonding configuration in those materials exhibiting nonthermal melting. This can be related to anharmonic coupling between phonon modes^33^,^34^. It has been shown that the transverse acoustic (TA) mode becomes unstable under high excitation in Si and InSb, leading to large bond distortion and eventually bond breaking to form disordered structure^32^,^33^,^34^. In this sense, the QES mediates an energy exchange from electronic to atomic degrees of freedom, transferring the mechanical work done by the electronic pressure to the kinetic energy of atoms. This is supported by the agreement between the observed roughly linear dependence of the lattice-collapse time constant on the laser fluence^15^ and the quantum Hooke's law, i.e., the linear dependence of QES on hole density. Of course, more detailed and thorough studies should be carried out in the future to correlate the microscopic processes and atomic dynamics, such as the TA phonon instability^32^,^33^,^34^ and fractional diffusion^35^, with the macroscopic theory of QES and quantum Hooke's law we present here.

We further point out that the QES induced crystal-to-liquid transition might not be the only transition in a given material under pulse laser irradiation, because there could be other high-pressure high-density crystalline solid phases competing with the liquid phase. The examples include pulse laser induced monoclinic-to-tetragonal phase transition in VO_2_^36^ and graphite-to-diamond phase transition in graphite^37^. However, there is a common feature associated with these transitions that the ending crystalline phase has a higher atomic density than the starting crystal phase, i.e.an anomalous solid-solid coexisting line in the T-P phase diagram^28^. Thus, we believe the QES is also the governing physical parameter in controlling such phase transitions^23^ and future theoretical studies may be done to explore this possibility.

In conclusion, we have revealed a fundamental mechanism underlying the pulse laser induced ultrafast crystal-to-liquid phase transitions, which are shown to be governed by the property of QES induced by electron-hole plasma that follows quantum Hooke's law. This enables us to classify such transitions into two distinct classes of materials: the ones undergoing ultrafast nonthermal melting process must occur in materials having an anomalous phase diagram with dT_m_/dP < 0; while those undergoing relatively slower thermal melting process occur preferably in all materials. A theoretical scheme is established for *a priori* prediction of materials to exhibit the ultrafast pulse laser induced crystal-to-liquid phase transitions, serving as a useful guide to future experiments.

## Author Contributions

F. L. originated the idea and directed the project. H. H. carried out most and H. D. carried out part of the theoretical calculations. F. L. and H. H. wrote the manuscript.

## Supplementary Material

Supplementary Information supplementary information

## Figures and Tables

**Figure 1:**
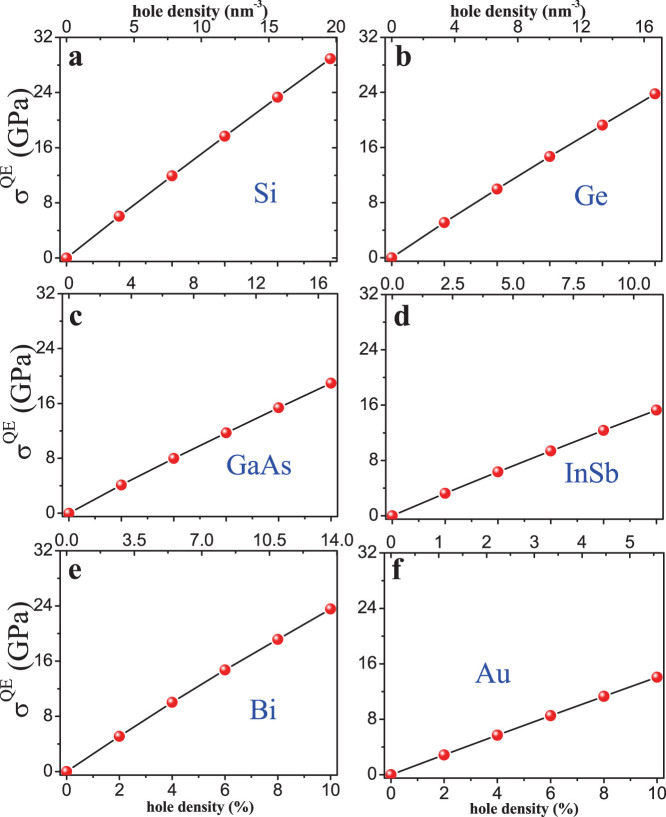
The QES induced by holes in different materials vs. hole density. The bottom x-axis labels the hole density in units of percentage of total valence electrons; the top x-axis in units of nm^-3^. (a) – (f) are for Si, Ge, GaAs, InSb, Bi and Au, respectively. We consider the outmost *sp* electrons as valence electron.

**Figure 2:**
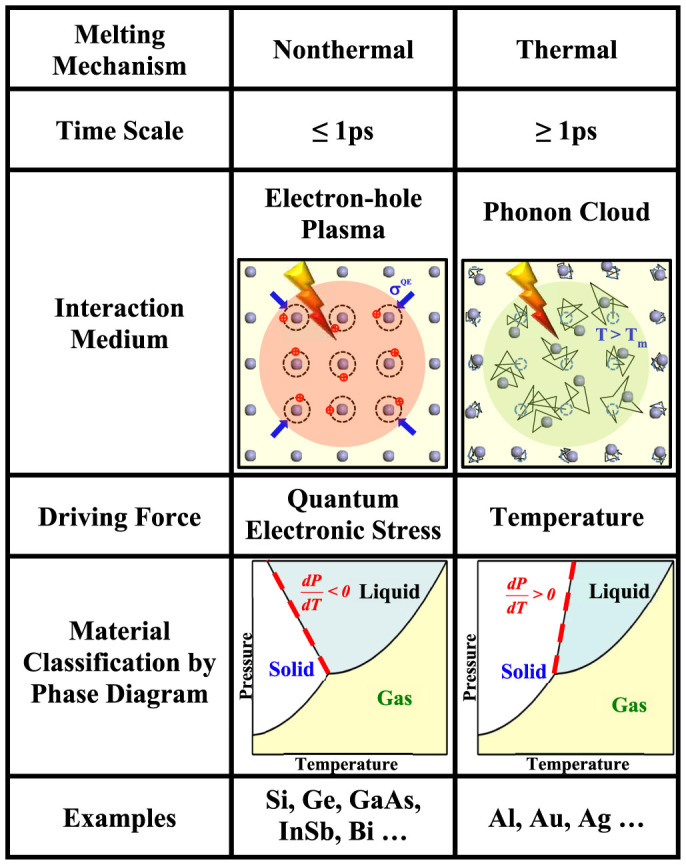
A table classifying the femtosecond pulse laser induced ultrafast crystal-to-liquid phase transitions, under high enough fluence. The distinct time scale, interaction medium, driving force, phase diagram and examples are listed to distinguish nonthermal from thermal melting process.

**Figure 3:**
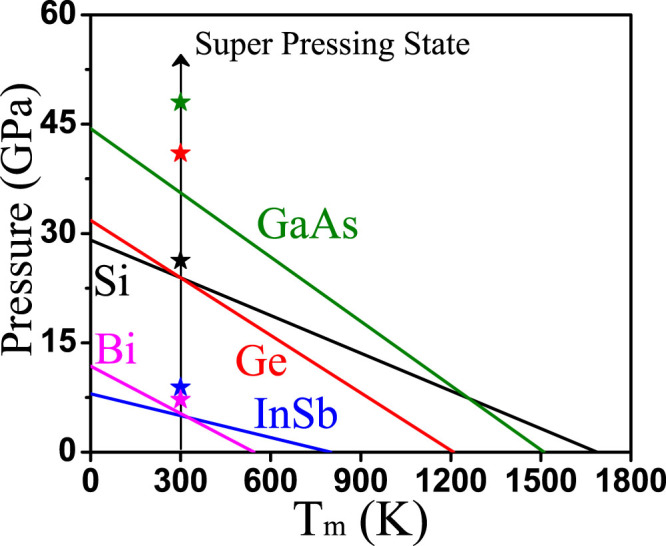
Comparison of the minimum pressure needed to reach "super pressing" state at room temperature with the QES at experimental threshold electron-hole plasma density. The QES at experimental threshold density are marked as stars. The electron-hole densities corresponding to the minimum pressure are 8.2%, 9.9%, 18.5%, 3.2% and 2.2% for Si, Ge, GaAs, InSb and Bi, respectively.

**Table 1:** Atomic Density (in unit of g/cm^-3^) and the slope of solid-liquid phase equilibrium line (dT_m_/dP, in unit of K/GPa) of known materials having an anomalous or normal phase diagram to exhibit nonthermal or thermal melting process under femtosecond pulse laser excitation, respectively. The data labeled ‘a’ are from Ref. 26, those labeled ‘b’ are from Ref. 27, those labeled ‘c’ are estimated from the phase diagram in Ref. 28, and the rest are from Wikipedia

	Substances	d_s_	d_l_	dT_m_/dP
Anomalous phase diagram d_s_ < d_l_	Si	2.329	2.57	−59^a^
	Ge	5.323	5.60	−38^a^
	GaAs	5.32	5.71^b^	−34^a^
	InSb	5.775	6.48^b^	−100^a^
	Bi	9.78	10.05	−46^c^
Normal phase diagram d_s_ > d_l_	Al	2.70	2.375	80^c^
	Au	19.30	17.31	57^c^
	Ag	10.49	9.32	44^c^

**Table 2:** Materials identified with anomalous phase diagram and predicted to exhibit nonthermal melting. Units are same as in Table I. P_m_ is the minimum pressure needed to reach "super pressing" state. The data are from Ref. 27

Substances	d_s_	d_l_	T_m_	dT_m_/dP	P_m_
AlSb	4.18	4.72	1353	−93	11.3
GaSb	5.60	6.06	985	−53	12.9
InAs	5.50	5.89	1215	−49	18.7
Mg_2_Si	1.84	2.27	1375	−129	8.3
Mg_2_Sn	3.45	3.52	1051	−22	34.1
Mg_2_Pb	5.00	5.20	823	−41	12.5

## References

[b1] Sundaram S. K. & Mazur E. Inducing and probing non-thermal transitions in semiconductors using femtosecond laser pulses. Nature Mater. 1, 217–224 (2002). 12618781 10.1038/nmat767

[b2] Siegal Y., Glezer E. N., Huang L. & Mazur E. Laser-induced phase transitions in semiconductors. Annu. Rev. Mater. Sci. 25, 223–247 (1995).

[b3] Van Vechten J. A., Tsu R. & Saris F. W. Nonthermal pulsed laser annealing of Si: plasma annealing. Phys. Lett. A 74, 422–426 (1979).

[b4] Shank C. V., Yen R. & Hirlimann C. Time-resolved reflectivity measurements of femtosecond-optical-pulse-induced phase transitions in silicon. Phys. Rev. Lett. 50, 454–457 (1983).

[b5] Tom H. W. K., Aumiller G. D. & Brito-Cruz C. H. Time-resolved study of laser-induced disorder of Si surfaces. Phys. Rev. Lett. 60, 1438–1441 (1988). 10038038 10.1103/PhysRevLett.60.1438

[b6] Saeta P., Wang J.-K., Siegal Y., Bloembergen N. & Mazur E. Ultrafast electronic disordering during femtosecond laser melting of GaAs. Phys. Rev. Lett. 67, 1023–1026 (1991). 10045050 10.1103/PhysRevLett.67.1023

[b7] Sokolowski-Tinten K., Schulz H., Bialkowski J. & von der Linde D. Two distinct transitions in ultrafast solid-liquid phase transformations of GaAs. Appl. Phys. A 53, 227–234 (1991).

[b8] Glezer E. N., Siegal Y., Huang L. & Mazur E. Behavior of χ^(2)^ during a laser-induced phase transition in GaAs. Phys. Rev. B 51, 9589–9596 (1995). 10.1103/physrevb.51.9589 9977621

[b9] Huang L., Callan J. P., Glezer E. N. & Mazur E. GaAs under intense ultrafast excitation: response of the dielectric function. Phys. Rev. Lett. 80, 185–188 (1998).

[b10] Siders C. W. *et al.* Detection of nonthermal melting by ultrafast X-ray diffraction. Science 286, 1340–1342 (1999). 10558985 10.1126/science.286.5443.1340

[b11] Harb M. *et al.* Electronically driven structure changes of Si captured by femtosecond electron diffraction. Phys. Rev. Lett. 100, 155504 (2008). 18518123 10.1103/PhysRevLett.100.155504

[b12] Shumay I. L. & Höfer U. Phase transformations of an InSb surface induced by strong femtosecond laser pulses. Phys. Rev. B 53, 15878–15884 (1996). 10.1103/physrevb.53.15878 9983426

[b13] Rousse A. *et al.* Non-thermal melting in semiconductors measured at femtosecond resolution. Nature 410, 65–68 (2001). 11242040 10.1038/35065045

[b14] Lindenberg A. M. *et al.* Atomic-scale visualization of inertial dynamics. Science 308, 392–395 (2005). 15831753 10.1126/science.1107996

[b15] Sciaini G. *et al.* Electronic acceleration of atomic motions and disordering in bismuth. Nature 458, 56–59 (2009). 19262668 10.1038/nature07788

[b16] Sokolowski-Tinten K. *et al.* Femtosecond X-ray measurement of coherent lattice vibrations near the Lindemann stability limit. Nature 422, 287–289 (2003). 12646915 10.1038/nature01490

[b17] Fritz D. M. *et al.* Ultrafast bond softening in Bismuth: mapping a solid's interatomic potential with X-rays. Science 315, 633–636 (2007). 17272718 10.1126/science.1135009

[b18] Johnson S. L. *et al.* Nanoscale depth-resolved coherent femtosecond motion in laser-excited Bismuth. Phys. Rev. Lett. 100, 155501 (2008). 18518120 10.1103/PhysRevLett.100.155501

[b19] Chen J., Chen W. –K., Tang, J. & Rentzepis, P. M. Time-resolved structural dynamics of thin metal films heated with femtosecond optical pulses. Proc. Natl. Acad. Sci. 108, 18887–18892 (2011). 22065752 10.1073/pnas.1115237108 PMC3223451

[b20] Siwick B. J., Dwyer J. R., Jordan R. E. & Dwayne Miller R. J. An atomic-level view of melting using femtosecond electron diffraction. Science 302, 1382–1385 (2003). 14631036 10.1126/science.1090052

[b21] Cao J. *et al.* Femtosecond electron diffraction for direct measurement of ultrafast atomic motions. Appl. Phys. Lett. 83, 1044–1046 (2003).

[b22] Wuttig M. & Yamada N. Phase-change materials for rewriteable data storage. Nature Mater. 6, 824–832 (2007). 17972937 10.1038/nmat2009

[b23] Hu H. *et al.* Quantum elec*t*ronic stress: density-functional-theory formulation and physical manifestation. Phys. Rev. Lett. 109, 055501 (2012). 23006185 10.1103/PhysRevLett.109.055501

[b24] Huber R. *et al.* How many-particle interactions develop after ultrafast excitation of an electron-hole plasma. Nature 414, 286–289 (2001). 11713523 10.1038/35104522

[b25] Levinshtein M., Rumyantsev S. & Shur M. Handbook Series on Semiconductor Parameters, Vol. 1 (World Scientific, 1996).

[b26] Jayaraman A., Klement, Jr W. & Kennedy G. C. Melting and polymorphism at high pressures in some group IV Elements and III-V compounds with the diamond/zincblende structure. Phys. Rev. 130, 540–547 (1963).

[b27] Glazov V. M., Chizhevskaya S. N. & Glagoleva N. N. Liquid Semiconductors (Plenum, New York, 1969).

[b28] Young D. A. Phase Diagram of the Elements (University of California Press, Oxford, England, 1991).

[b29] Chen J., Chen W.-K. & Rentzepis M. R. Blast wave and contraction in Au (111) thin film induced by femtosecond laser pulses. A time resolved x-ray diffraction study. J. Appl. Phys. 109, 113522 (2011).

[b30] Silvestrelli P. L., Alavi A., Parrinello M. & Frenkel D. *Ab initio* molecular dynamics simulation of laser melting of silicon. Phys. Rev. Lett. 77, 3149–3152 (1996). 10062146 10.1103/PhysRevLett.77.3149

[b31] Shick A. B., Ketterson J. B., Novikov D. L. & Freeman A. J. Electronic structure, phase stability, and semimetal-semiconductor transitions in Bi. Phys. Rev. B 60, 15484 (1999).

[b32] Stampfli P. & Bennemann K. H. Theory for the laser-induced femtosecond phase transition of silicon and GaAs. Appl. Phys. A 60, 191–196 (1995).

[b33] Zijlstra E. S., Walkenhorst J. & Garcia M. E. Anharmonic noninertial lattice dynamics during ultrafast nonthermal melting of InSb. Phys. Rev. Lett. 101, 135701 (2008). 18851461 10.1103/PhysRevLett.101.135701

[b34] Recoules V., Clerouin J., Zerah G., Anglade P. M. & Mazevet S. Effect of intense laser irradiation on the lattice stability of semiconductors and metals. Phys. Rev. Lett. 96, 055503 (2006). 16486947 10.1103/PhysRevLett.96.055503

[b35] Zijlstra E. S., Kalitsov A., Zier T. & Garcia M. E. Fractional diffusion in Silicon. Adv. Mater. 25, 5605–5608 (2013). 23925994 10.1002/adma201302559

[b36] Cavalleri A. *et al.* Femtosecond structural dynamics in VO_2_ during an ultrafast solid-solid phase transition. Phys. Rev. Lett. 87, 237401 (2001). 11736474 10.1103/PhysRevLett.87.237401

[b37] Kanasaki J., Inami E., Tanimura K., Ohnishi H. & Nasu K. Formation of *sp^3^*-bonded carbon nanostructures by femtosecond laser excitation of graphite. Phys. Rev. Lett. 102, 087402 (2009). 19257783 10.1103/PhysRevLett.102.087402

