# Supplementary material for: Quantum Hooke's Law to Classify Pulse Laser Induced Ultrafast Melting
Source: Sci Rep. 2015 Feb 3;5:8212. doi: 10.1038/srep08212 (PMC4314650; doi:10.1038/srep08212)
Supplement: Supplementary Information [file srep08212-s1.pdf]

Supplementary Information

Quantum Hooke's Law to Classify Pulse Laser Induced Ultrafast Melting

Hao Hu[1,2], Hepeng Ding[2] and Feng Liu[2,*]

[1]Frontier Institute of Science and Technology, Xi'an Jiaotong University, Xi'an 710054, China

[2]Department of Materials Science and Engineering, University of Utah, Salt Lake City, UT 84112, USA

1. First principle calculation of hole-induced quantum electronic stress (QES)

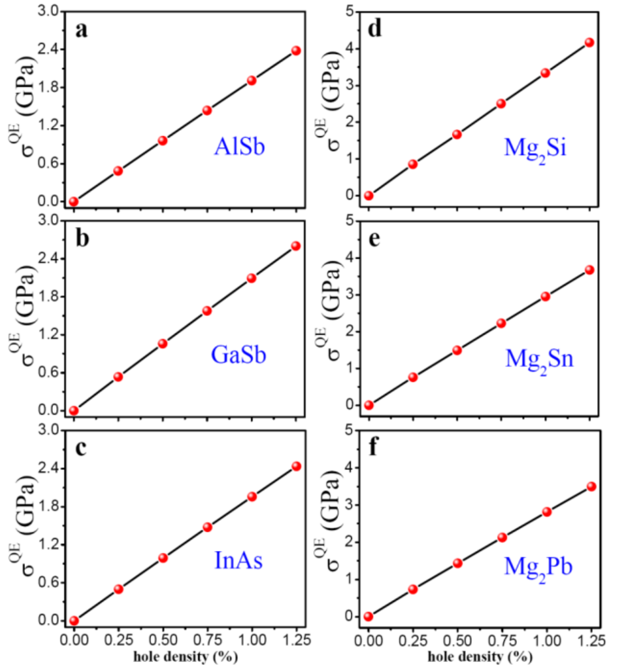

Figure S1. The hole induced QES in the predicted nonthermal melting materials.

The first-principles calculations are based on the density functional theory plane-wave method as implemented in Vienna ab initio simulation package (VASP)[1]. In calculating bulk stress induced by charge carriers, the projector augmented wave (PAW) method[2] and the PBE exchange-correlation functionals[3] are used. Tests have been done with respect to plane-wave energy cutoff and k-point sampling for each system to ensure convergence. The QES are obtained by calculating

the difference between the Martin-Nielsen mechanical stress tensor[4] at the excited or perturbed electron density and that at the ground-state density[5]. The values we show in Fig. 1 in the main text are the average of the three diagonal components of the stress tensor, i.e. $\sigma = (\sigma_{xx} + \sigma_{yy} + \sigma_{zz})/3$. For cubic lattice, the QES induced by hole is isotropic, i.e. $\sigma_{xx}=\sigma_{yy}=\sigma_{zz}$; while for other lattice, the QES could be anisotropic.

Similarly, figure S1 shows the hole induced QES in the materials that we proposed in Table II in the main text.

2. QES induced by a homogeneous electron gas at T = 0 K

For a homogeneous electron gas, QES is equal to the degeneracy pressure (DP) coming from the Pauli exclusion of electrons[6]. The DP can be calculated from the derivative of total energy of the homogeneous electron gas with respect to the volume change. We use a numerical model to quantify the QES of homogeneous electron gas. The total energy of homogeneous gas can be expressed as

$$E = V \times \varepsilon(\rho) \times \rho, \qquad (1)$$

where V is volume, ρ is the electron density and ε(ρ) is the total energy per electron of homogeneous electron gas with density ρ. It contains terms of kinetic energy, exchange energy and correlation energy,

$$\varepsilon(\rho) = \varepsilon_T(\rho) + \varepsilon_x(\rho) + \varepsilon_c(\rho). \qquad (2)$$

The kinetic energy and exchange energy at T = 0 K can be expressed analytically:

$$\varepsilon_T = \frac{3}{10}\left(3\pi^2\right)^{2/3} \rho^{2/3}, \qquad (3a)$$

$$\varepsilon_x = -\frac{3}{4}\left(\frac{3}{\pi}\right)^{1/3} \rho^{1/3} . \qquad (3b)$$

Atomic units are used throughout here unless otherwise specified. Usually for a homogeneous electron gas, another variable r_s is used instead of ρ with

$$\frac{4}{3}\pi r_s^3 = \frac{1}{\rho} , \qquad (4)$$

so the total energy per electron can be expressed as

$$\varepsilon\left(r_s\right) = \frac{1.1049}{r_s^2} - \frac{0.4582}{r_s} + \varepsilon_c\left(r_s\right) . \qquad (5)$$

In general, there is no analytic expression for $\varepsilon_c(r_s)$, so it is calculated numerically. We use the VWN interpolation formula for $\varepsilon_c(r_s)$, which gives very good results in the density range we are interested in[7]:

$$\varepsilon_c\left(r_s\right) = A\left\{\ln\frac{x^2}{X(x)} + \frac{2b}{Q}\arctan\frac{Q}{2x+b} - \frac{bx_0}{X(x_0)}\left[\ln\frac{\left(x-x_0\right)^2}{X\left(x\right)} + \frac{2\left(b+2x_0\right)}{Q}\right]\arctan\frac{Q}{2x+b}\right\}, \qquad (6)$$

where $x = \sqrt{r_s}$, $X\left(x\right) = x^2 + bx + c$, and $Q = \sqrt{4c - b^2}$. The parameters A=0.0621814, x_0=-0.10498, b=3.72744, c=12.9352 give the best fitting to Ceperley and Alder's quantum Monte Carlo results[8].

The QES of homogeneous electron gas can be calculated straightforwardly as

$$\sigma^{QE} = -P = \frac{dU}{dV} . \qquad (7)$$

The negative sign convention means that a positive tensile QES (equivalent to a negative internal pressure) tends to shrink the volume, while a negative compressive QES (a positive internal pressure) tends to expand the volume.

Figure S2 shows the DP induced by different energy terms as well as the total pressure. One sees that the kinetic energy always induces positive pressure (P_T), tending to expand the volume; while both the exchange and correlation energy induce negative pressure (P_x and P_c), tending to shrink the volume. At very low electron density, the exchange and correlation terms dominate (increasing faster with the increasing density); at high density, the kinetic term dominates. This gives rise to a minimum total pressure within the electron density range of our interest (0 – 20 nm^{-3} as shown in Fig. 1 in the main text). The inset shows the total pressure in the density range of 0 – 20 nm^{-3}, which has a minimum at relatively low electron density, and its magnitude stays less than 1 GPa. This means that the QES of electron gas in the electron-hole plasma is essentially negligible compared to the QES induced by the localized valence holes. Therefore, we can approximate the QES induced the electron-hole plasma by just the QES induced by holes as shown in Fig. 1 in the main text.

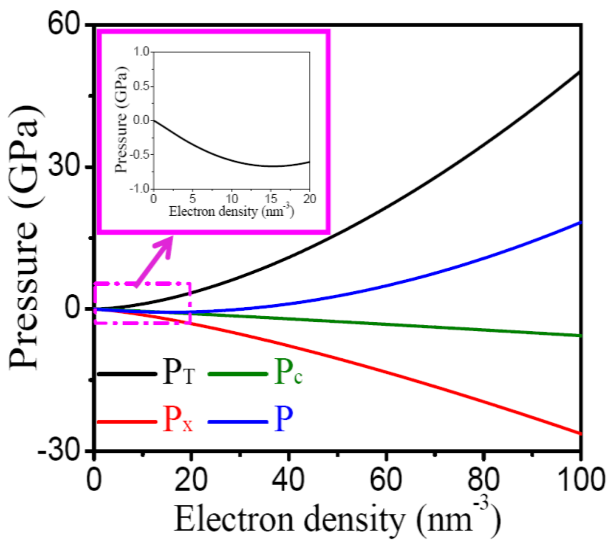

Figure S2. DP induced by different energy terms and the total pressure vs. electron density. Inset shows the total DP for an interacting homogeneous electron gas (P) and that for a free electron gas (P_T) in the density range of 0 – 20 nm^{-3}.

3. QES of a free electron gas at finite temperature

When the electron are excited by the pulse laser, it possess high kinetic energy (several eV), the temperature of the electron system could be very high, tens of thousands kelvin. At finite temperature, the kinetic energy, exchange and correlation energy vary with temperature, and so does the DP. Here, for simplicity, we only consider the kinetic term, i.e. we calculate the temperature dependent DP of a free electron gas, to see how the temperature may affect the QES.

For a free electron gas, the density of states is

$$D(\varepsilon) = \frac{V}{2\pi^2}\left(\frac{2m}{\hbar^2}\right)^{3/2} \varepsilon^{1/2}, \quad (8)$$

at finite temperature, the electrons obey Fermi-Dirac distribution, the total energy of the free electron gas can be expressed as

$$E_{tot} = \int_0^\infty \varepsilon f(\varepsilon) D(\varepsilon) d\varepsilon = \frac{V}{2\pi^2}\left(\frac{2m}{\hbar^2}\right)^{3/2} \int_0^\infty \frac{\varepsilon^{3/2}}{e^{(\varepsilon-\mu(T))/k_B T}+1} d\varepsilon, \quad (9)$$

the electron number can also be expressed by the integral as

$$N = \int_0^\infty f(\varepsilon) D(\varepsilon) d\varepsilon = \frac{V}{2\pi^2}\left(\frac{2m}{\hbar^2}\right)^{3/2} \int_0^\infty \frac{\varepsilon^{1/2}}{e^{(\varepsilon-\mu(T))/k_B T}+1} d\varepsilon, \quad (10)$$

where V is the volume, μ is the chemical potential of the free electron gas, which varies with temperature. To calculate the total energy vs. T, we need to calculate μ(T) first.

At T = 0 K, the electron number

$$N = \frac{V}{2\pi^2}\left(\frac{2m}{\hbar^2}\right)^{3/2} \int_0^{\varepsilon_F} \varepsilon^{1/2} d\varepsilon = \frac{V}{2\pi^2}\left(\frac{2m}{\hbar^2}\right)^{3/2} \frac{2}{3}\varepsilon_F^{3/2}, \tag{11}$$

where ε_F is Fermi energy.

Since the electron number does not change with temperature, we have

$$\frac{2}{3}\varepsilon_F^{3/2} = \int_0^\infty \frac{\varepsilon^{1/2}}{e^{(\varepsilon-\mu)/k_BT}+1} d\varepsilon, \tag{12}$$

Equation (12) can be expressed in a dimensionless form as

$$\frac{2}{3} = \int_0^\infty \frac{x^{1/2}}{e^{(x-\tilde{\mu})/\tilde{T}}+1} dx, \tag{13}$$

where $\tilde{\mu} = \mu/\varepsilon_F$, $\tilde{T} = T/T_F$, and $T_F = \varepsilon_F/k_B$ is Fermi temperature. Using Eq. (13), we can calculate the chemical potential vs. temperature numerically, as shown in Fig. S3.

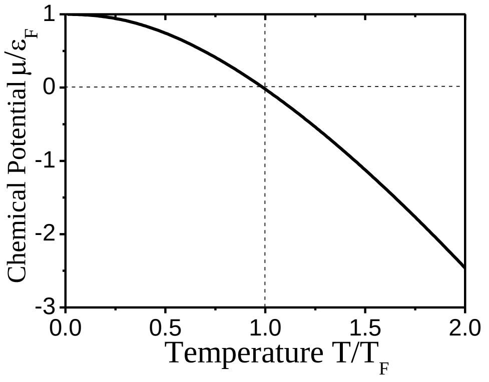

Figure S3. Chemical potential of free electron gas vs. temperature.

At any given electron density, we can calculate the Fermi energy from Eq. (11) as

$$\varepsilon_F = \frac{\hbar^2}{2m}\left(3\pi^2\rho\right)^{2/3}. \tag{14}$$

Using Eq. (9), (13) and (14), we can calculate the total energy of free electron gas at given electron density and temperature, numerically. The DP of free electron gas at finite temperature can be calculated by

$$P = -\frac{dE_{tot}}{dV} . \qquad (15)$$

Figure S4 shows the calculated DP vs. electron density at different temperature. We can see that only at very high temperature ($>10^4$ K), the DP differs notably from that at zero temperature; the DP decreases with increasing temperature, which is probably due to the decreasing of chemical potential with increasing temperature, so that adding electron into the system (increasing electron density) increases less energy to the total energy. Another way we may understand such behavior is that at higher temperature, the electrons distribute quasi-equally in all energy states($<k_BT$), so the Pauli exclusion effect becomes weak, and so does the DP.

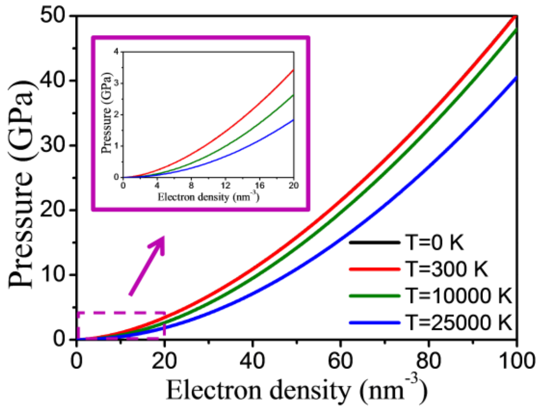

Figure S4. DP of free electron gas vs. electron density at different temperature. The two curves of T = 0 K (black) and T = 300 K (red) are almost identical.

Reference:

1. Kresse, G. & Hafner, J. Ab initio molecular dynamics for liquid metals. *Phys. Rev. B* **47**, 558-561 (1993).
2. Blöchl, P. E. Projector augmented-wave method. *Phys. Rev. B* **50**, 17953-17979 (1994).
3. Perdew, J. P., Burke, K. & Ernzerhof, M. Generalized Gradient Approximation made simple. *Phys. Rev. Let*t. **77**, 3865-3868 (1996).
4. Nielsen, O. H. & Martin, R. M. First-principles calculation of stress. *Phys. Rev. Lett.* **50**, 697-700 (1983); Quantum-mechanical theory of stress and force. *Phys. Rev. B* **32**, 3780-3791 (1985).
5. Hu, H. *et al*. Quantum electronic stress: density-functional-theory formulation and physical manifestation. *Phys. Rev. Lett.* **109**, 055501 (2012).
6. Hu, H. & Liu, F. Density-functional-theory formulation of classical and quantum Hooke's Law. *Science China: Technological Sciences* **57**, 692-698 (2014).
7. Vosko, S. H., Wilk, L. & Nusair, M. Accurate spin-dependent electron liquid correlation energies for local spin density calculations: a critical analysis. *Can. J. Phys.* **58**, 1200-1211 (1980).
8. Ceperley, D. M. & Alder, B. J. Ground state of the electron gas by a stochastic method. *Phys. Rev. Lett.* **45**, 566-569 (1980).
